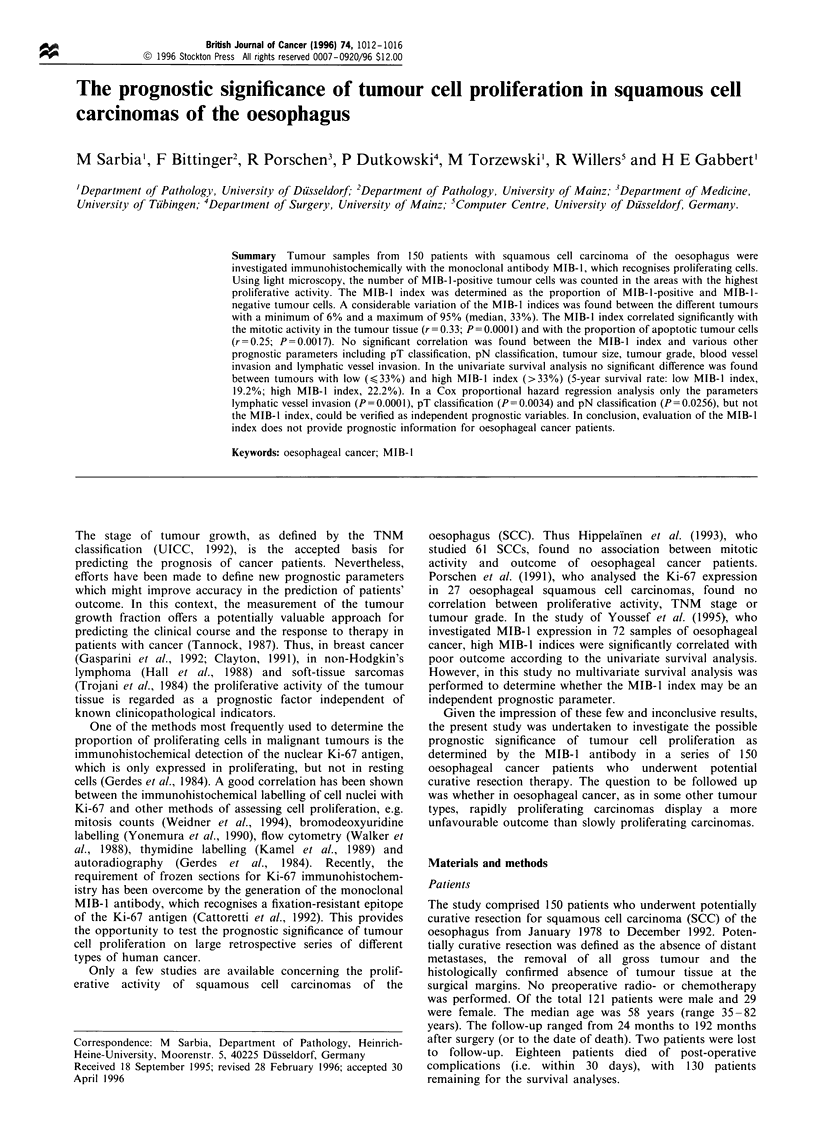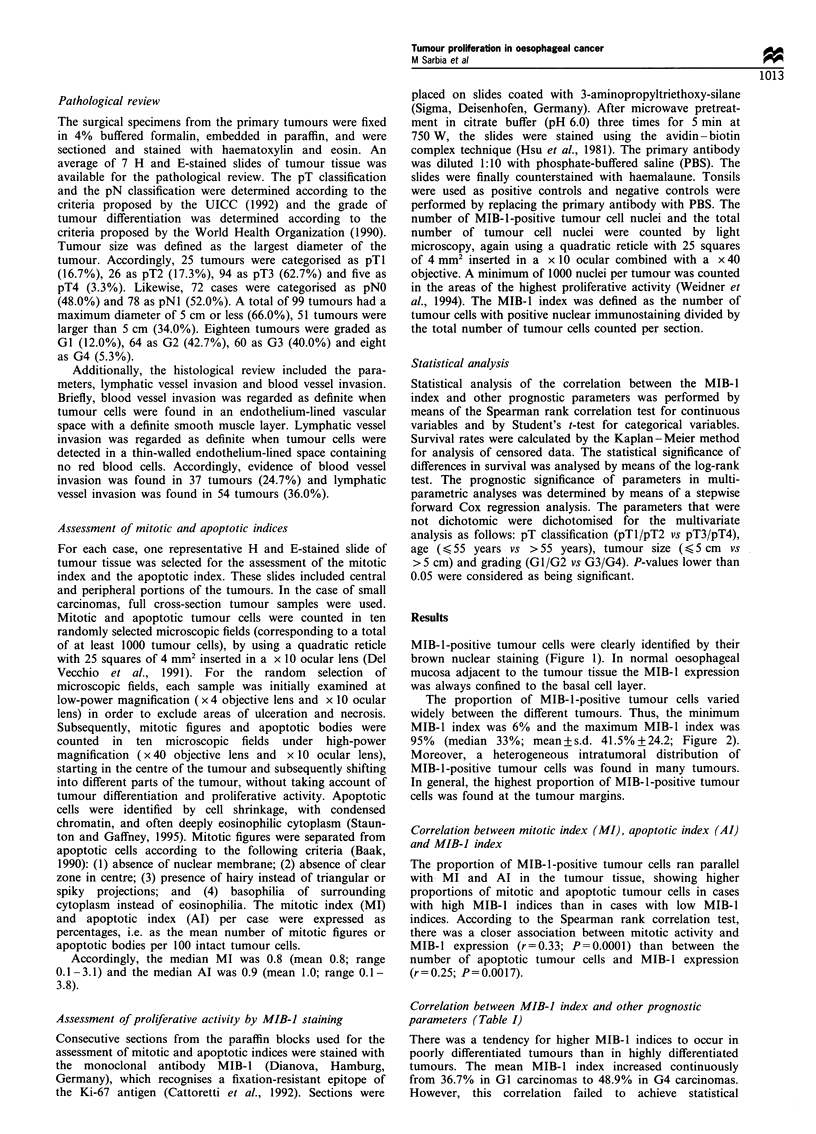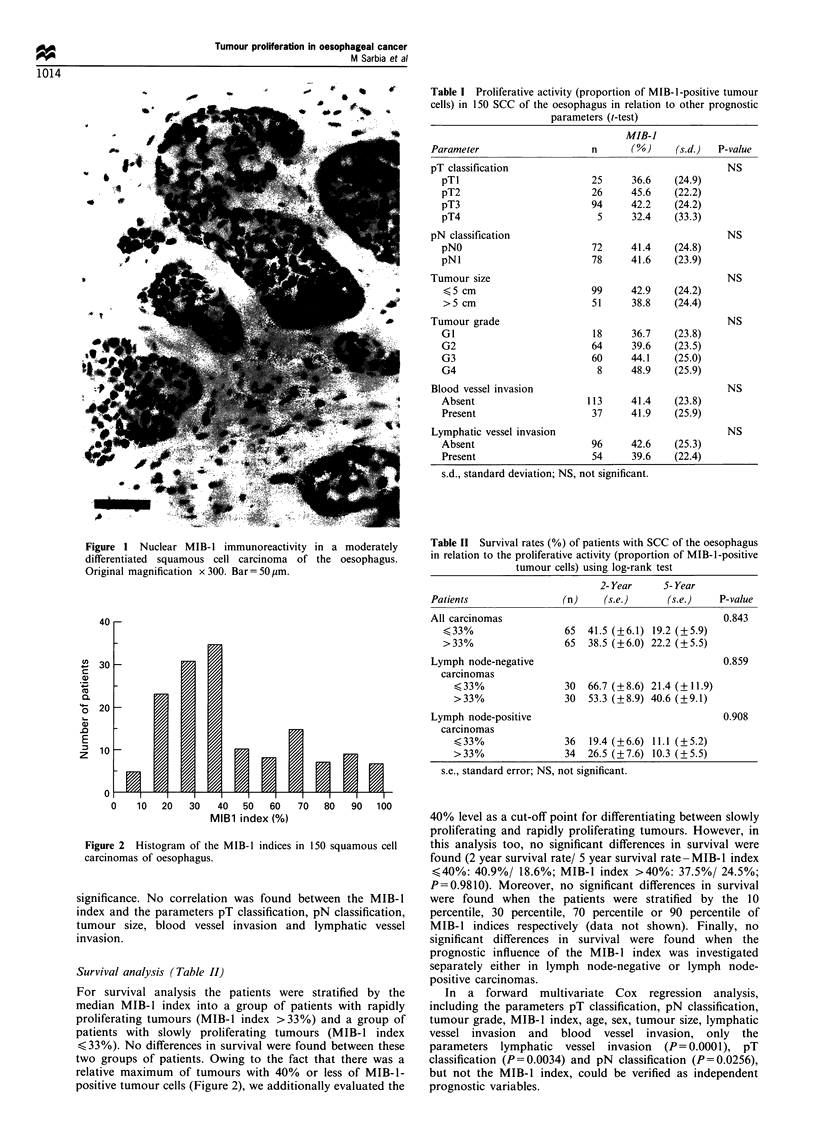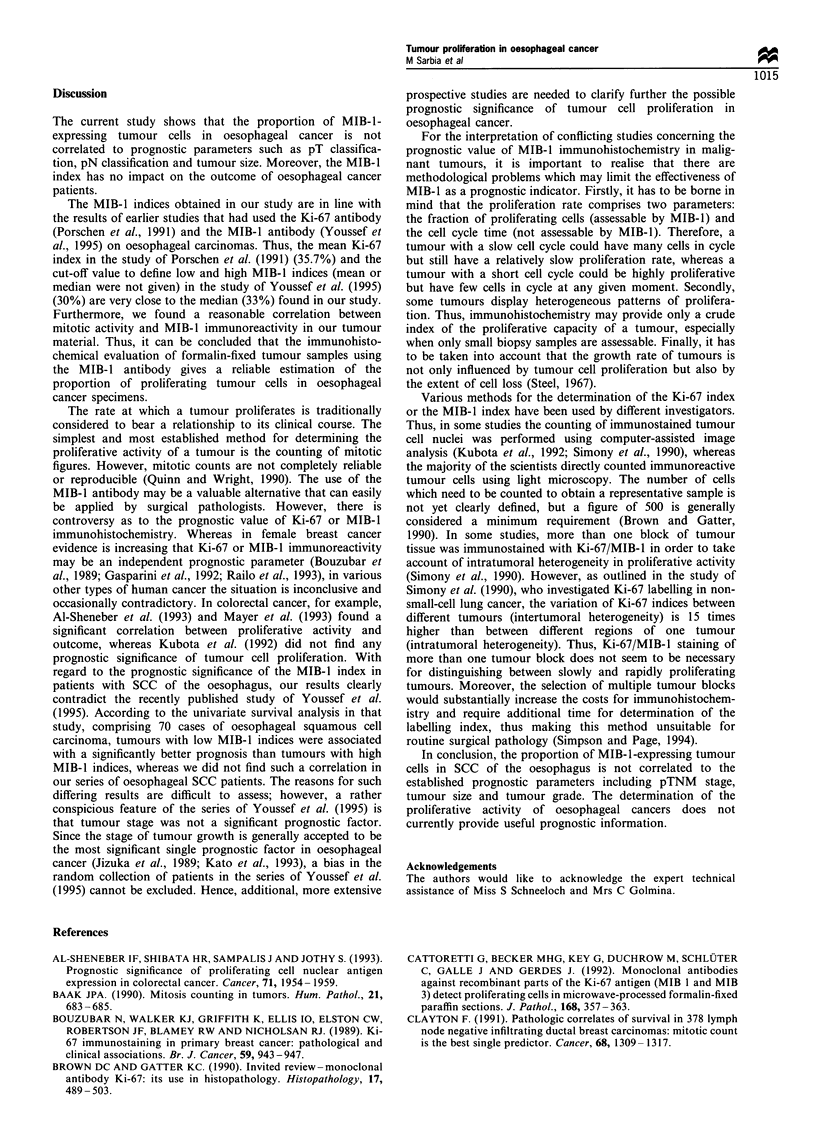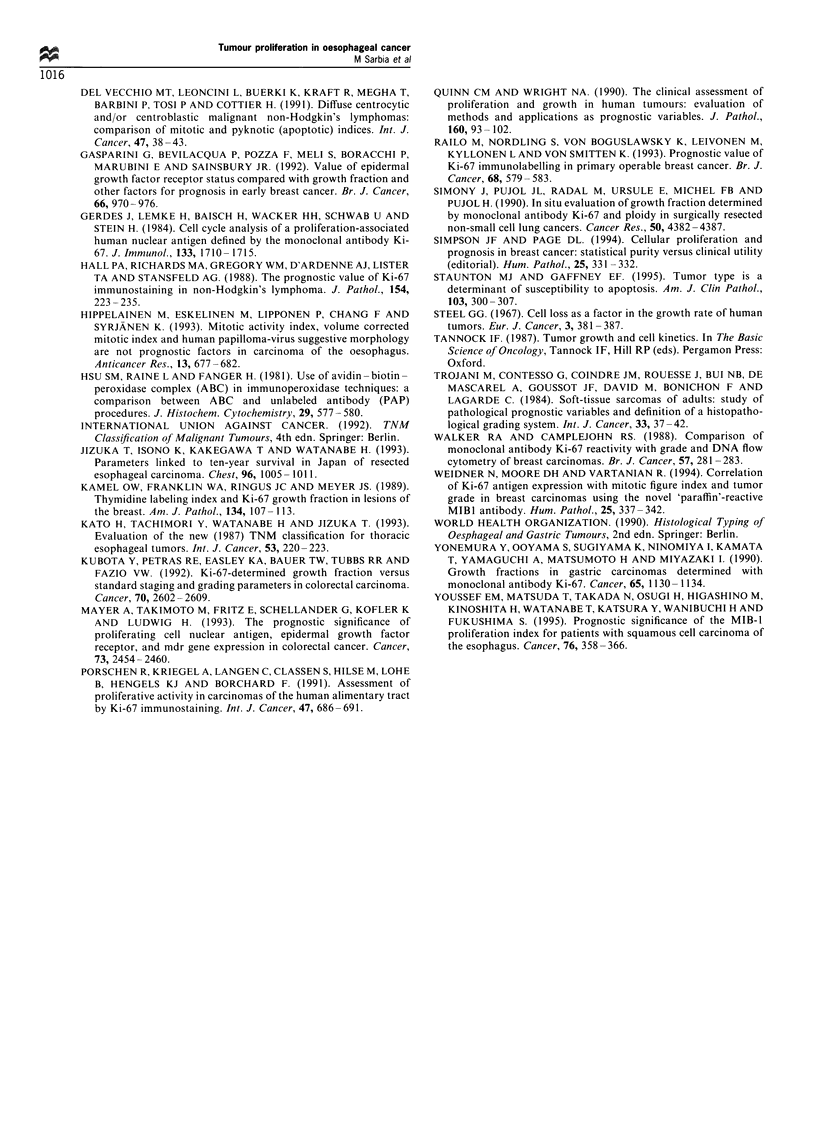# The prognostic significance of tumour cell proliferation in squamous cell carcinomas of the oesophagus.

**DOI:** 10.1038/bjc.1996.482

**Published:** 1996-10

**Authors:** M. Sarbia, F. Bittinger, R. Porschen, P. Dutkowski, M. Torzewski, R. Willers, H. E. Gabbert

**Affiliations:** Department of Pathology, University of Düsseldorf, Germany.

## Abstract

**Images:**


British Journal of Cancer (1996) 74, 1012-1016
© 1996 Stockton Press All rights reserved 0007-0920/96 $12.00

### The prognostic significance of tumour cell proliferation in squamous cell carcinomas of the oesophagus

M Sarbia[1], F Bittinger[2], R Porschen[3], P Dutkowski[4], M Torzewski[1], R Willers[5] and H E Gabbert[1]

[1]*Department of Pathology, University of Düsseldorf;* [2]*Department of Pathology, University of Mainz;* [3]*Department of Medicine, University of Tübingen;* [4]*Department of Surgery, University of Mainz;* [5]*Computer Centre, University of Düsseldorf, Germany.*

**Summary** Tumour samples from 150 patients with squamous cell carcinoma of the oesophagus were investigated immunohistochemically with the monoclonal antibody MIB-1, which recognises proliferating cells. Using light microscopy, the number of MIB-1-positive tumour cells was counted in the areas with the highest proliferative activity. The MIB-1 index was determined as the proportion of MIB-1-positive and MIB-1-negative tumour cells. A considerable variation of the MIB-1 indices was found between the different tumours with a minimum of 6% and a maximum of 95% (median, 33%). The MIB-1 index correlated significantly with the mitotic activity in the tumour tissue ($r=0.33$; $P=0.0001$) and with the proportion of apoptotic tumour cells ($r=0.25$; $P=0.0017$). No significant correlation was found between the MIB-1 index and various other prognostic parameters including pT classification, pN classification, tumour size, tumour grade, blood vessel invasion and lymphatic vessel invasion. In the univariate survival analysis no significant difference was found between tumours with low ($\leqslant 33\%$) and high MIB-1 index ($>33\%$) (5-year survival rate: low MIB-1 index, 19.2%; high MIB-1 index, 22.2%). In a Cox proportional hazard regression analysis only the parameters lymphatic vessel invasion ($P=0.0001$), pT classification ($P=0.0034$) and pN classification ($P=0.0256$), but not the MIB-1 index, could be verified as independent prognostic variables. In conclusion, evaluation of the MIB-1 index does not provide prognostic information for oesophageal cancer patients.

**Keywords:** oesophageal cancer; MIB-1

The stage of tumour growth, as defined by the TNM classification (UICC, 1992), is the accepted basis for predicting the prognosis of cancer patients. Nevertheless, efforts have been made to define new prognostic parameters which might improve accuracy in the prediction of patients' outcome. In this context, the measurement of the tumour growth fraction offers a potentially valuable approach for predicting the clinical course and the response to therapy in patients with cancer (Tannock, 1987). Thus, in breast cancer (Gasparini *et al.*, 1992; Clayton, 1991), in non-Hodgkin's lymphoma (Hall *et al.*, 1988) and soft-tissue sarcomas (Trojani *et al.*, 1984) the proliferative activity of the tumour tissue is regarded as a prognostic factor independent of known clinicopathological indicators.

One of the methods most frequently used to determine the proportion of proliferating cells in malignant tumours is the immunohistochemical detection of the nuclear Ki-67 antigen, which is only expressed in proliferating, but not in resting cells (Gerdes *et al.*, 1984). A good correlation has been shown between the immunohistochemical labelling of cell nuclei with Ki-67 and other methods of assessing cell proliferation, e.g. mitosis counts (Weidner *et al.*, 1994), bromodeoxyuridine labelling (Yonemura *et al.*, 1990), flow cytometry (Walker *et al.*, 1988), thymidine labelling (Kamel *et al.*, 1989) and autoradiography (Gerdes *et al.*, 1984). Recently, the requirement of frozen sections for Ki-67 immunohistochemistry has been overcome by the generation of the monoclonal MIB-1 antibody, which recognises a fixation-resistant epitope of the Ki-67 antigen (Cattoretti *et al.*, 1992). This provides the opportunity to test the prognostic significance of tumour cell proliferation on large retrospective series of different types of human cancer.

Only a few studies are available concerning the proliferative activity of squamous cell carcinomas of the oesophagus (SCC). Thus Hippelaïnen *et al.* (1993), who studied 61 SCCs, found no association between mitotic activity and outcome of oesophageal cancer patients. Porschen *et al.* (1991), who analysed the Ki-67 expression in 27 oesophageal squamous cell carcinomas, found no correlation between proliferative activity, TNM stage or tumour grade. In the study of Youssef *et al.* (1995), who investigated MIB-1 expression in 72 samples of oesophageal cancer, high MIB-1 indices were significantly correlated with poor outcome according to the univariate survival analysis. However, in this study no multivariate survival analysis was performed to determine whether the MIB-1 index may be an independent prognostic parameter.

Given the impression of these few and inconclusive results, the present study was undertaken to investigate the possible prognostic significance of tumour cell proliferation as determined by the MIB-1 antibody in a series of 150 oesophageal cancer patients who underwent potential curative resection therapy. The question to be followed up was whether in oesophageal cancer, as in some other tumour types, rapidly proliferating carcinomas display a more unfavourable outcome than slowly proliferating carcinomas.

### Materials and methods

### *Patients*

The study comprised 150 patients who underwent potentially curative resection for squamous cell carcinoma (SCC) of the oesophagus from January 1978 to December 1992. Potentially curative resection was defined as the absence of distant metastases, the removal of all gross tumour and the histologically confirmed absence of tumour tissue at the surgical margins. No preoperative radio- or chemotherapy was performed. Of the total 121 patients were male and 29 were female. The median age was 58 years (range 35–82 years). The follow-up ranged from 24 months to 192 months after surgery (or to the date of death). Two patients were lost to follow-up. Eighteen patients died of post-operative complications (i.e. within 30 days), with 130 patients remaining for the survival analyses.

Correspondence: M Sarbia, Department of Pathology, Heinrich-Heine-University, Moorenstr. 5, 40225 Düsseldorf, Germany
Received 18 September 1995; revised 28 February 1996; accepted 30 April 1996

*Pathological review*

The surgical specimens from the primary tumours were fixed in 4% buffered formalin, embedded in paraffin, and were sectioned and stained with haematoxylin and eosin. An average of 7 H and E-stained slides of tumour tissue was available for the pathological review. The pT classification and the pN classification were determined according to the criteria proposed by the UICC (1992) and the grade of tumour differentiation was determined according to the criteria proposed by the World Health Organization (1990). Tumour size was defined as the largest diameter of the tumour. Accordingly, 25 tumours were categorised as pT1 (16.7%), 26 as pT2 (17.3%), 94 as pT3 (62.7%) and five as pT4 (3.3%). Likewise, 72 cases were categorised as pN0 (48.0%) and 78 as pN1 (52.0%). A total of 99 tumours had a maximum diameter of 5 cm or less (66.0%), 51 tumours were larger than 5 cm (34.0%). Eighteen tumours were graded as G1 (12.0%), 64 as G2 (42.7%), 60 as G3 (40.0%) and eight as G4 (5.3%).

Additionally, the histological review included the parameters, lymphatic vessel invasion and blood vessel invasion. Briefly, blood vessel invasion was regarded as definite when tumour cells were found in an endothelium-lined vascular space with a definite smooth muscle layer. Lymphatic vessel invasion was regarded as definite when tumour cells were detected in a thin-walled endothelium-lined space containing no red blood cells. Accordingly, evidence of blood vessel invasion was found in 37 tumours (24.7%) and lymphatic vessel invasion was found in 54 tumours (36.0%).

*Assessment of mitotic and apoptotic indices*

For each case, one representative H and E-stained slide of tumour tissue was selected for the assessment of the mitotic index and the apoptotic index. These slides included central and peripheral portions of the tumours. In the case of small carcinomas, full cross-section tumour samples were used. Mitotic and apoptotic tumour cells were counted in ten randomly selected microscopic fields (corresponding to a total of at least 1000 tumour cells), by using a quadratic reticle with 25 squares of 4 $mm^2$ inserted in a $\times 10$ ocular lens (Del Vecchio *et al.*, 1991). For the random selection of microscopic fields, each sample was initially examined at low-power magnification ($\times 4$ objective lens and $\times 10$ ocular lens) in order to exclude areas of ulceration and necrosis. Subsequently, mitotic figures and apoptotic bodies were counted in ten microscopic fields under high-power magnification ($\times 40$ objective lens and $\times 10$ ocular lens), starting in the centre of the tumour and subsequently shifting into different parts of the tumour, without taking account of tumour differentiation and proliferative activity. Apoptotic cells were identified by cell shrinkage, with condensed chromatin, and often deeply eosinophilic cytoplasm (Staunton and Gaffney, 1995). Mitotic figures were separated from apoptotic cells according to the following criteria (Baak, 1990): (1) absence of nuclear membrane; (2) absence of clear zone in centre; (3) presence of hairy instead of triangular or spiky projections; and (4) basophilia of surrounding cytoplasm instead of eosinophilia. The mitotic index (MI) and apoptotic index (AI) per case were expressed as percentages, i.e. as the mean number of mitotic figures or apoptotic bodies per 100 intact tumour cells.

Accordingly, the median MI was 0.8 (mean 0.8; range 0.1–3.1) and the median AI was 0.9 (mean 1.0; range 0.1–3.8).

*Assessment of proliferative activity by MIB-1 staining*

Consecutive sections from the paraffin blocks used for the assessment of mitotic and apoptotic indices were stained with the monoclonal antibody MIB-1 (Dianova, Hamburg, Germany), which recognises a fixation-resistant epitope of the Ki-67 antigen (Cattoretti *et al.*, 1992). Sections were placed on slides coated with 3-aminopropyltriethoxy-silane (Sigma, Deisenhofen, Germany). After microwave pretreatment in citrate buffer (pH 6.0) three times for 5 min at 750 W, the slides were stained using the avidin–biotin complex technique (Hsu *et al.*, 1981). The primary antibody was diluted 1:10 with phosphate-buffered saline (PBS). The slides were finally counterstained with haemalaune. Tonsils were used as positive controls and negative controls were performed by replacing the primary antibody with PBS. The number of MIB-1-positive tumour cell nuclei and the total number of tumour cell nuclei were counted by light microscopy, again using a quadratic reticle with 25 squares of 4 $mm^2$ inserted in a $\times 10$ ocular combined with a $\times 40$ objective. A minimum of 1000 nuclei per tumour was counted in the areas of the highest proliferative activity (Weidner *et al.*, 1994). The MIB-1 index was defined as the number of tumour cells with positive nuclear immunostaining divided by the total number of tumour cells counted per section.

*Statistical analysis*

Statistical analysis of the correlation between the MIB-1 index and other prognostic parameters was performed by means of the Spearman rank correlation test for continuous variables and by Student's *t*-test for categorical variables. Survival rates were calculated by the Kaplan–Meier method for analysis of censored data. The statistical significance of differences in survival was analysed by means of the log-rank test. The prognostic significance of parameters in multiparametric analyses was determined by means of a stepwise forward Cox regression analysis. The parameters that were not dichotomic were dichotomised for the multivariate analysis as follows: pT classification (pT1/pT2 *vs* pT3/pT4), age ($\leqslant 55$ years *vs* $>55$ years), tumour size ($\leqslant 5$ cm *vs* $>5$ cm) and grading (G1/G2 *vs* G3/G4). *P*-values lower than 0.05 were considered as being significant.

### Results

MIB-1-positive tumour cells were clearly identified by their brown nuclear staining (Figure 1). In normal oesophageal mucosa adjacent to the tumour tissue the MIB-1 expression was always confined to the basal cell layer.

The proportion of MIB-1-positive tumour cells varied widely between the different tumours. Thus, the minimum MIB-1 index was 6% and the maximum MIB-1 index was 95% (median 33%; mean±s.d. 41.5%±24.2; Figure 2). Moreover, a heterogeneous intratumoral distribution of MIB-1-positive tumour cells was found in many tumours. In general, the highest proportion of MIB-1-positive tumour cells was found at the tumour margins.

*Correlation between mitotic index (MI), apoptotic index (AI) and MIB-1 index*

The proportion of MIB-1-positive tumour cells ran parallel with MI and AI in the tumour tissue, showing higher proportions of mitotic and apoptotic tumour cells in cases with high MIB-1 indices than in cases with low MIB-1 indices. According to the Spearman rank correlation test, there was a closer association between mitotic activity and MIB-1 expression ($r=0.33$; $P=0.0001$) than between the number of apoptotic tumour cells and MIB-1 expression ($r=0.25$; $P=0.0017$).

*Correlation between MIB-1 index and other prognostic parameters (Table I)*

There was a tendency for higher MIB-1 indices to occur in poorly differentiated tumours than in highly differentiated tumours. The mean MIB-1 index increased continuously from 36.7% in G1 carcinomas to 48.9% in G4 carcinomas. However, this correlation failed to achieve statistical

**Figure 1** Nuclear MIB-1 immunoreactivity in a moderately differentiated squamous cell carcinoma of the oesophagus. Original magnification ×300. Bar = 50 μm.

**Figure 2** Histogram of the MIB-1 indices in 150 squamous cell carcinomas of oesophagus.

significance. No correlation was found between the MIB-1 index and the parameters pT classification, pN classification, tumour size, blood vessel invasion and lymphatic vessel invasion.

*Survival analysis (Table II)*

For survival analysis the patients were stratified by the median MIB-1 index into a group of patients with rapidly proliferating tumours (MIB-1 index >33%) and a group of patients with slowly proliferating tumours (MIB-1 index ⩽33%). No differences in survival were found between these two groups of patients. Owing to the fact that there was a relative maximum of tumours with 40% or less of MIB-1-positive tumour cells (Figure 2), we additionally evaluated the 40% level as a cut-off point for differentiating between slowly proliferating and rapidly proliferating tumours. However, in this analysis too, no significant differences in survival were found (2 year survival rate/ 5 year survival rate – MIB-1 index ⩽40%: 40.9%/ 18.6%; MIB-1 index >40%: 37.5%/ 24.5%; $P = 0.9810$). Moreover, no significant differences in survival were found when the patients were stratified by the 10 percentile, 30 percentile, 70 percentile or 90 percentile of MIB-1 indices respectively (data not shown). Finally, no significant differences in survival were found when the prognostic influence of the MIB-1 index was investigated separately either in lymph node-negative or lymph node-positive carcinomas.

In a forward multivariate Cox regression analysis, including the parameters pT classification, pN classification, tumour grade, MIB-1 index, age, sex, tumour size, lymphatic vessel invasion and blood vessel invasion, only the parameters lymphatic vessel invasion ($P = 0.0001$), pT classification ($P = 0.0034$) and pN classification ($P = 0.0256$), but not the MIB-1 index, could be verified as independent prognostic variables.

**Table I** Proliferative activity (proportion of MIB-1-positive tumour cells) in 150 SCC of the oesophagus in relation to other prognostic parameters (*t*-test)

| *Parameter* | n | *MIB-1 (%)* | *(s.d.)* | P-*value* |
|---|---|---|---|---|
| pT classification | | | | NS |
| pT1 | 25 | 36.6 | (24.9) | |
| pT2 | 26 | 45.6 | (22.2) | |
| pT3 | 94 | 42.2 | (24.2) | |
| pT4 | 5 | 32.4 | (33.3) | |
| pN classification | | | | NS |
| pN0 | 72 | 41.4 | (24.8) | |
| pN1 | 78 | 41.6 | (23.9) | |
| Tumour size | | | | NS |
| ⩽5 cm | 99 | 42.9 | (24.2) | |
| >5 cm | 51 | 38.8 | (24.4) | |
| Tumour grade | | | | NS |
| G1 | 18 | 36.7 | (23.8) | |
| G2 | 64 | 39.6 | (23.5) | |
| G3 | 60 | 44.1 | (25.0) | |
| G4 | 8 | 48.9 | (25.9) | |
| Blood vessel invasion | | | | NS |
| Absent | 113 | 41.4 | (23.8) | |
| Present | 37 | 41.9 | (25.9) | |
| Lymphatic vessel invasion | | | | NS |
| Absent | 96 | 42.6 | (25.3) | |
| Present | 54 | 39.6 | (22.4) | |

s.d., standard deviation; NS, not significant.

**Table II** Survival rates (%) of patients with SCC of the oesophagus in relation to the proliferative activity (proportion of MIB-1-positive tumour cells) using log-rank test

| *Patients* | *(n)* | *2-Year (s.e.)* | *5-Year (s.e.)* | P-*value* |
|---|---|---|---|---|
| All carcinomas | | | | 0.843 |
| ⩽33% | 65 | 41.5 (±6.1) | 19.2 (±5.9) | |
| >33% | 65 | 38.5 (±6.0) | 22.2 (±5.5) | |
| Lymph node-negative carcinomas | | | | 0.859 |
| ⩽33% | 30 | 66.7 (±8.6) | 21.4 (±11.9) | |
| >33% | 30 | 53.3 (±8.9) | 40.6 (±9.1) | |
| Lymph node-positive carcinomas | | | | 0.908 |
| ⩽33% | 36 | 19.4 (±6.6) | 11.1 (±5.2) | |
| >33% | 34 | 26.5 (±7.6) | 10.3 (±5.5) | |

s.e., standard error; NS, not significant.

### Discussion

The current study shows that the proportion of MIB-1-expressing tumour cells in oesophageal cancer is not correlated to prognostic parameters such as pT classification, pN classification and tumour size. Moreover, the MIB-1 index has no impact on the outcome of oesophageal cancer patients.

The MIB-1 indices obtained in our study are in line with the results of earlier studies that had used the Ki-67 antibody (Porschen *et al.*, 1991) and the MIB-1 antibody (Youssef *et al.*, 1995) on oesophageal carcinomas. Thus, the mean Ki-67 index in the study of Porschen *et al.* (1991) (35.7%) and the cut-off value to define low and high MIB-1 indices (mean or median were not given) in the study of Youssef *et al.* (1995) (30%) are very close to the median (33%) found in our study. Furthermore, we found a reasonable correlation between mitotic activity and MIB-1 immunoreactivity in our tumour material. Thus, it can be concluded that the immunohistochemical evaluation of formalin-fixed tumour samples using the MIB-1 antibody gives a reliable estimation of the proportion of proliferating tumour cells in oesophageal cancer specimens.

The rate at which a tumour proliferates is traditionally considered to bear a relationship to its clinical course. The simplest and most established method for determining the proliferative activity of a tumour is the counting of mitotic figures. However, mitotic counts are not completely reliable or reproducible (Quinn and Wright, 1990). The use of the MIB-1 antibody may be a valuable alternative that can easily be applied by surgical pathologists. However, there is controversy as to the prognostic value of Ki-67 or MIB-1 immunohistochemistry. Whereas in female breast cancer evidence is increasing that Ki-67 or MIB-1 immunoreactivity may be an independent prognostic parameter (Bouzubar *et al.*, 1989; Gasparini *et al.*, 1992; Railo *et al.*, 1993), in various other types of human cancer the situation is inconclusive and occasionally contradictory. In colorectal cancer, for example, Al-Sheneber *et al.* (1993) and Mayer *et al.* (1993) found a significant correlation between proliferative activity and outcome, whereas Kubota *et al.* (1992) did not find any prognostic significance of tumour cell proliferation. With regard to the prognostic significance of the MIB-1 index in patients with SCC of the oesophagus, our results clearly contradict the recently published study of Youssef *et al.* (1995). According to the univariate survival analysis in that study, comprising 70 cases of oesophageal squamous cell carcinoma, tumours with low MIB-1 indices were associated with a significantly better prognosis than tumours with high MIB-1 indices, whereas we did not find such a correlation in our series of oesophageal SCC patients. The reasons for such differing results are difficult to assess; however, a rather conspicious feature of the series of Youssef *et al.* (1995) is that tumour stage was not a significant prognostic factor. Since the stage of tumour growth is generally accepted to be the most significant single prognostic factor in oesophageal cancer (Jizuka *et al.*, 1989; Kato *et al.*, 1993), a bias in the random collection of patients in the series of Youssef *et al.* (1995) cannot be excluded. Hence, additional, more extensive prospective studies are needed to clarify further the possible prognostic significance of tumour cell proliferation in oesophageal cancer.

For the interpretation of conflicting studies concerning the prognostic value of MIB-1 immunohistochemistry in malignant tumours, it is important to realise that there are methodological problems which may limit the effectiveness of MIB-1 as a prognostic indicator. Firstly, it has to be borne in mind that the proliferation rate comprises two parameters: the fraction of proliferating cells (assessable by MIB-1) and the cell cycle time (not assessable by MIB-1). Therefore, a tumour with a slow cell cycle could have many cells in cycle but still have a relatively slow proliferation rate, whereas a tumour with a short cell cycle could be highly proliferative but have few cells in cycle at any given moment. Secondly, some tumours display heterogeneous patterns of proliferation. Thus, immunohistochemistry may provide only a crude index of the proliferative capacity of a tumour, especially when only small biopsy samples are assessable. Finally, it has to be taken into account that the growth rate of tumours is not only influenced by tumour cell proliferation but also by the extent of cell loss (Steel, 1967).

Various methods for the determination of the Ki-67 index or the MIB-1 index have been used by different investigators. Thus, in some studies the counting of immunostained tumour cell nuclei was performed using computer-assisted image analysis (Kubota *et al.*, 1992; Simony *et al.*, 1990), whereas the majority of the scientists directly counted immunoreactive tumour cells using light microscopy. The number of cells which need to be counted to obtain a representative sample is not yet clearly defined, but a figure of 500 is generally considered a minimum requirement (Brown and Gatter, 1990). In some studies, more than one block of tumour tissue was immunostained with Ki-67/MIB-1 in order to take account of intratumoral heterogeneity in proliferative activity (Simony *et al.*, 1990). However, as outlined in the study of Simony *et al.* (1990), who investigated Ki-67 labelling in non-small-cell lung cancer, the variation of Ki-67 indices between different tumours (intertumoral heterogeneity) is 15 times higher than between different regions of one tumour (intratumoral heterogeneity). Thus, Ki-67/MIB-1 staining of more than one tumour block does not seem to be necessary for distinguishing between slowly and rapidly proliferating tumours. Moreover, the selection of multiple tumour blocks would substantially increase the costs for immunohistochemistry and require additional time for determination of the labelling index, thus making this method unsuitable for routine surgical pathology (Simpson and Page, 1994).

In conclusion, the proportion of MIB-1-expressing tumour cells in SCC of the oesophagus is not correlated to the established prognostic parameters including pTNM stage, tumour size and tumour grade. The determination of the proliferative activity of oesophageal cancers does not currently provide useful prognostic information.

**Acknowledgements**
The authors would like to acknowledge the expert technical assistance of Miss S Schneeloch and Mrs C Golmina.

### References

AL-SHENEBER IF, SHIBATA HR, SAMPALIS J AND JOTHY S. (1993). Prognostic significance of proliferating cell nuclear antigen expression in colorectal cancer. *Cancer*, **71**, 1954–1959.

BAAK JPA. (1990). Mitosis counting in tumors. *Hum. Pathol.*, **21**, 683–685.

BOUZUBAR N, WALKER KJ, GRIFFITH K, ELLIS IO, ELSTON CW, ROBERTSON JF, BLAMEY RW AND NICHOLSAN RJ. (1989). Ki-67 immunostaining in primary breast cancer: pathological and clinical associations. *Br. J. Cancer*, **59**, 943–947.

BROWN DC AND GATTER KC. (1990). Invited review–monoclonal antibody Ki-67: its use in histopathology. *Histopathology*, **17**, 489–503.

CATTORETTI G, BECKER MHG, KEY G, DUCHROW M, SCHLÜTER C, GALLE J AND GERDES J. (1992). Monoclonal antibodies against recombinant parts of the Ki-67 antigen (MIB 1 and MIB 3) detect proliferating cells in microwave-processed formalin-fixed paraffin sections. *J. Pathol.*, **168**, 357–363.

CLAYTON F. (1991). Pathologic correlates of survival in 378 lymph node negative infiltrating ductal breast carcinomas: mitotic count is the best single predictor. *Cancer*, **68**, 1309–1317.

DEL VECCHIO MT, LEONCINI L, BUERKI K, KRAFT R, MEGHA T, BARBINI P, TOSI P AND COTTIER H. (1991). Diffuse centrocytic and/or centroblastic malignant non-Hodgkin's lymphomas: comparison of mitotic and pyknotic (apoptotic) indices. *Int. J. Cancer*, **47**, 38–43.

GASPARINI G, BEVILACQUA P, POZZA F, MELI S, BORACCHI P, MARUBINI E AND SAINSBURY JR. (1992). Value of epidermal growth factor receptor status compared with growth fraction and other factors for prognosis in early breast cancer. *Br. J. Cancer*, **66**, 970–976.

GERDES J, LEMKE H, BAISCH H, WACKER HH, SCHWAB U AND STEIN H. (1984). Cell cycle analysis of a proliferation-associated human nuclear antigen defined by the monoclonal antibody Ki-67. *J. Immunol.*, **133**, 1710–1715.

HALL PA, RICHARDS MA, GREGORY WM, D'ARDENNE AJ, LISTER TA AND STANSFELD AG. (1988). The prognostic value of Ki-67 immunostaining in non-Hodgkin's lymphoma. *J. Pathol.*, **154**, 223–235.

HIPPELAINEN M, ESKELINEN M, LIPPONEN P, CHANG F AND SYRJÄNEN K. (1993). Mitotic activity index, volume corrected mitotic index and human papilloma-virus suggestive morphology are not prognostic factors in carcinoma of the oesophagus. *Anticancer Res.*, **13**, 677–682.

HSU SM, RAINE L AND FANGER H. (1981). Use of avidin–biotin–peroxidase complex (ABC) in immunoperoxidase techniques: a comparison between ABC and unlabeled antibody (PAP) procedures. *J. Histochem. Cytochemistry*, **29**, 577–580.

INTERNATIONAL UNION AGAINST CANCER. (1992). *TNM Classification of Malignant Tumours*, 4th edn. Springer: Berlin.

JIZUKA T, ISONO K, KAKEGAWA T AND WATANABE H. (1993). Parameters linked to ten-year survival in Japan of resected esophageal carcinoma. *Chest*, **96**, 1005–1011.

KAMEL OW, FRANKLIN WA, RINGUS JC AND MEYER JS. (1989). Thymidine labeling index and Ki-67 growth fraction in lesions of the breast. *Am. J. Pathol.*, **134**, 107–113.

KATO H, TACHIMORI Y, WATANABE H AND JIZUKA T. (1993). Evaluation of the new (1987) TNM classification for thoracic esophageal tumors. *Int. J. Cancer*, **53**, 220–223.

KUBOTA Y, PETRAS RE, EASLEY KA, BAUER TW, TUBBS RR AND FAZIO VW. (1992). Ki-67-determined growth fraction versus standard staging and grading parameters in colorectal carcinoma. *Cancer*, **70**, 2602–2609.

MAYER A, TAKIMOTO M, FRITZ E, SCHELLANDER G, KOFLER K AND LUDWIG H. (1993). The prognostic significance of proliferating cell nuclear antigen, epidermal growth factor receptor, and mdr gene expression in colorectal cancer. *Cancer*, **73**, 2454–2460.

PORSCHEN R, KRIEGEL A, LANGEN C, CLASSEN S, HILSE M, LOHE B, HENGELS KJ AND BORCHARD F. (1991). Assessment of proliferative activity in carcinomas of the human alimentary tract by Ki-67 immunostaining. *Int. J. Cancer*, **47**, 686–691.

QUINN CM AND WRIGHT NA. (1990). The clinical assessment of proliferation and growth in human tumours: evaluation of methods and applications as prognostic variables. *J. Pathol.*, **160**, 93–102.

RAILO M, NORDLING S, VON BOGUSLAWSKY K, LEIVONEN M, KYLLONEN L AND VON SMITTEN K. (1993). Prognostic value of Ki-67 immunolabelling in primary operable breast cancer. *Br. J. Cancer*, **68**, 579–583.

SIMONY J, PUJOL JL, RADAL M, URSULE E, MICHEL FB AND PUJOL H. (1990). In situ evaluation of growth fraction determined by monoclonal antibody Ki-67 and ploidy in surgically resected non-small cell lung cancers. *Cancer Res.*, **50**, 4382–4387.

SIMPSON JF AND PAGE DL. (1994). Cellular proliferation and prognosis in breast cancer: statistical purity versus clinical utility (editorial). *Hum. Pathol.*, **25**, 331–332.

STAUNTON MJ AND GAFFNEY EF. (1995). Tumor type is a determinant of susceptibility to apoptosis. *Am. J. Clin Pathol.*, **103**, 300–307.

STEEL GG. (1967). Cell loss as a factor in the growth rate of human tumors. *Eur. J. Cancer*, **3**, 381–387.

TANNOCK IF. (1987). Tumor growth and cell kinetics. In *The Basic Science of Oncology*, Tannock IF, Hill RP (eds). Pergamon Press: Oxford.

TROJANI M, CONTESSO G, COINDRE JM, ROUESSE J, BUI NB, DE MASCAREL A, GOUSSOT JF, DAVID M, BONICHON F AND LAGARDE C. (1984). Soft-tissue sarcomas of adults: study of pathological prognostic variables and definition of a histopathological grading system. *Int. J. Cancer*, **33**, 37–42.

WALKER RA AND CAMPLEJOHN RS. (1988). Comparison of monoclonal antibody Ki-67 reactivity with grade and DNA flow cytometry of breast carcinomas. *Br. J. Cancer*, **57**, 281–283.

WEIDNER N, MOORE DH AND VARTANIAN R. (1994). Correlation of Ki-67 antigen expression with mitotic figure index and tumor grade in breast carcinomas using the novel 'paraffin'-reactive MIB1 antibody. *Hum. Pathol.*, **25**, 337–342.

WORLD HEALTH ORGANIZATION. (1990). *Histological Typing of Oesphageal and Gastric Tumours*, 2nd edn. Springer: Berlin.

YONEMURA Y, OOYAMA S, SUGIYAMA K, NINOMIYA I, KAMATA T, YAMAGUCHI A, MATSUMOTO H AND MIYAZAKI I. (1990). Growth fractions in gastric carcinomas determined with monoclonal antibody Ki-67. *Cancer*, **65**, 1130–1134.

YOUSSEF EM, MATSUDA T, TAKADA N, OSUGI H, HIGASHINO M, KINOSHITA H, WATANABE T, KATSURA Y, WANIBUCHI H AND FUKUSHIMA S. (1995). Prognostic significance of the MIB-1 proliferation index for patients with squamous cell carcinoma of the esophagus. *Cancer*, **76**, 358–366.